# A case report of infectious scleritis with corneal ulcer caused by *Scedosporium aurantiacum*

**DOI:** 10.1097/MD.0000000000016063

**Published:** 2019-07-05

**Authors:** Hyuna Kim, Ja-Young Ahn, In-Young Chung, Seong-Wook Seo, Woong-Sun Yoo, Jong Hee Shin, Seong-Jae Kim

**Affiliations:** a Department of Ophthalmology, Gyeongsang National University Hospital; b Department of Ophthalmology, School of Medicine and Institute of Health Science, Gyeongsang National University, Jinju; c Department of Laboratory Medicine, Chonnam National University Medical School, Gwangju, Republic of Korea.

**Keywords:** enucleation, infectious keratitis, *Scedosporium aurantiacum*

## Abstract

**Rationale:**

*Scedosporium* species is rare pathogen of ocular infection. The accurate diagnosis is delaying in many cases and the clinical prognosis is poor due to its resistance to antifungal agents. This report describes a patient with infectious scleritis and corneal ulcer caused by *Scedosporium auranticum* infection who required enucleation to control the infection.

**Patient concerns:**

A 70-year-old woman visited our clinic after experiencing ocular discomfort in her right eye for 4 days after minor ocular trauma, with soil exposure.

**Diagnoses:**

*Scedosporium* species was isolated from a culture of corneal tissue, *Scedosporium aurantiacum* was identified in a culture of necrotic tissue.

**Interventions:**

She was started on treatment with antifungal agents, including topical amphotericin B and systemic fluconazole, but her ocular condition did not improve. Although the lesion showed temporary improvement, ocular pain and corneal ulcer recurred 3 months later. Evisceration was performed due to corneal perforation, and enucleation was also performed for dehiscence of the conjunctiva and scleral necrosis.

**Outcomes:**

After enucleation, postoperative systemic voriconazole treatment controlled the infection without recurrence.

**Lessons:**

*S aurantiacum* keratitis is difficult to eradicate, even with several months of treatment with systemic and topical antifungal agents, and tends to progress to scleritis. The infection can be terminated by the orbital enucleation. Infection with this rare organism should be included in the differential diagnosis of patients with severe infectious keratitis.

## 1 Introduction

Fungi of the genus *Scedosporium* are ubiquitous filamentous fungi commonly found in soil, decaying vegetation, manure, multiple water sources, standing water, and potted plants in hospitals.^[1]^ These organisms have been identified as emerging opportunistic pathogens responsible for fungal infections in immunocompromised and occasionally in immunocompetent patients. These organisms can infect any part of the human body, especially the parasinus, lungs, soft tissue, central nervous system, and bone.^[2]^ Keratitis caused by *Scedosporium* species is rare, delaying accurate diagnoses. Patients infected with these organisms have a poor clinical prognosis because these fungi are usually resistant to conventional antifungal agents. The species of *Scedosporium* most frequently identified in infected patients is *Scedosporium apiospermum*.

This report describes a patient with infectious scleritis and corneal ulcer caused by *Scedosporium auranticum* infection who required enucleation to control the infection. To our knowledge, this is the first description of *Scedosporium aurantiacum* keratitis in an immunocompetent patient with only well-controlled hypertension. The patient has provided informed consent for this study. The review of patient charts providing data for this study was approved by the Institutional Review Board of the Gyeongsang National University Hospital (2012-09-003-001).

## 2 Case report

**Figure 1:**
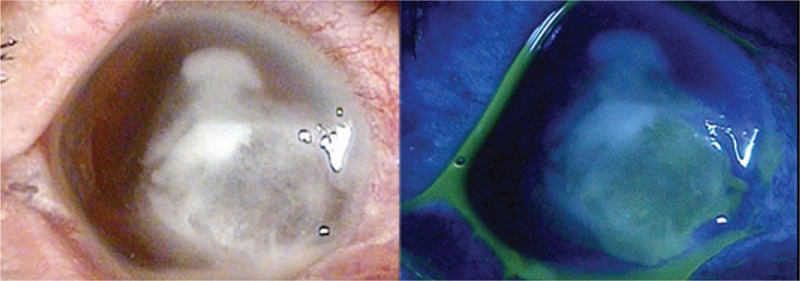
Photograph of the anterior segment of the right eye of the patient at initial presentation, showing a corneal epithelial defect, stromal infiltration, melting, thinning, and hypopyon.

A 70-year-old woman was referred to our hospital due to ocular discomfort in her right eye. Her symptoms had started 10 days earlier and coincided with a mild eye injury due to soil. At that time, she was seen at an ophthalmology clinic and was treated with topical moxifloxacin 0.5% (Vigamox, Alcon, TX) and ofloxacin (Quinovid, Hanlim, Seoul, South Korea) for 1 week. Because her condition subsequently deteriorated, she was referred to our tertiary ophthalmology clinic. She had been diagnosed with hypertension 7 years earlier, but did not have any other relevant ocular history. Upon examination, her visual acuity was hand motion in the right eye and 20/25 in the left eye. Slit lamp examination showed a marked conjunctival infection with epithelial defects and large stromal infiltration with corneal melting. The anterior chamber was filled with inflammatory cells and a 1.5 mm hypopyon level (Fig. 1). The posterior chamber appeared hazy. The intraocular pressure (IOP) in her right eye was 10 mmHg, and the IOP in her left eye was 15 mmHg.

Corneal scrapings were taken immediately and cultured on blood agar, chocolate agar, Sabaroud dextrose agar (SDA), and eosin methylene blue agar. A Potassium Hydroxide (KOH) wet preparation test revealed the presence of fungal hyphae. These microscopic findings, along with the soil exposure history of the patient and her lack of response to antibiotics, suggested fungal keratitis. She was started on topical moxifloxacin 0.5% (Moroxacin, Hanmi, Seoul, South Korea), 0.2% fluconazole (Diflucan, Pfizer, Cedex, France), and 0.125% amphotericin B (Fungizone, BMS, South Korea) eye drops every 30 minutes, as well as intravenous systemic fluconazole (Diflucan, Pfizer, Cedex, France) 100 mg twice daily. After 4 days, the size of the corneal epithelial defect and infiltration had increased and the edema had worsened. A new satellite lesion appeared superotemporal to the original lesion, and the amount of hypopyon had increased to a height of 2.0 mm. The following day, a species of *Scedosporium* was isolated from the first corneal scraping culture. Because *Scedosporium* species are more susceptible to voriconazole than to other antifungal agents, the patient was started on treatment with topical voriconazle 2% (Vfend, Pfizer, Sandwich, UK) eye drops, as well as 4 mg/kg systemic intravenous voriconazole (Vfend), administered twice daily. Although the corneal epithelial defect and corneal melting slightly improved and corneal thinning did not progress, anterior chamber inflammation and hypopyon persisted. On day 16, the patient was discharged on treatment with topical moxifloxacin 0.5% every 2 hours, 0.125% amphotericin B and voriconazole 2% every hour, and 200 mg systemic voriconazole (PO) every 12 hours.

**Figure 2:**
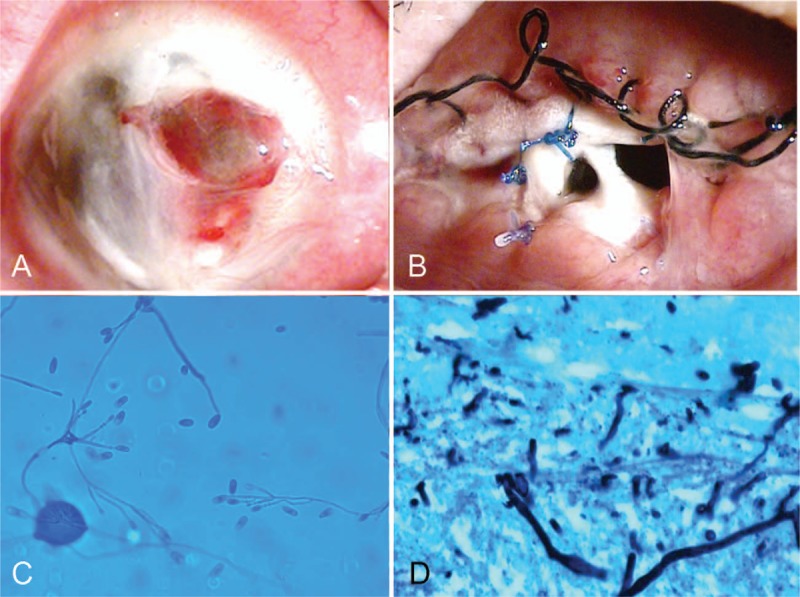
Photographs of the anterior segment of the right eye after 3 months of treatment, showing (A) corneal melting and perforation, (B) conjunctival wound dehiscence, a scleral knot and silicone ball exposure, scleral melting, and necrosis. (C, D) isolated organism, identified as *Scedosporium aurantiacum*.

Examination 1 month later showed that her corneal lesion had stabilized. Her treatment was changed, with eye drops instilled every 4 hours and systemic voriconazole dosage reduced. However, at 3 months, ocular pain recurred in her right eye. Slit lamp examination showed that the area of corneal stromal infiltration and hypopyon had increased, corneal melting was advanced, and a corneal perforation had occurred (Fig. 2A). After deciding to remove the infected tissue surgically, the patient underwent evisceration with silicone ball implantation, followed by intravenous administration of 200 mg ciprofloxacin (Citopcin, CJ, Seoul, South Korea) twice daily, and, after 4 days of pressure pad dressing, instillation of moxifloxacin 0.5% eye drops every 4 hours. The patient was discharged 1 week later with a stabilized conjunctival sutured site.

Two weeks later, dehiscence of the conjunctiva and scleral was observed, for which the patient underwent additional suturing of the conjunctiva and sclera, and amniotic membrane implantation. Ten days after the second operation, the patient presented with amniotic membrane melting and re-dehiscence of tissues, along with necrotic scleritis (Fig. 2B). An orbital computed tomography (CT) scan showed thickening of the entire sclera and enhancement of the orbit. To control the recurrent regional infection, an additional enucleation was performed.

The fungal isolate was obtained from the necrotic tissue, and it was identified as *Scedosporium aurantiacum* by macroscopic and microscopic morphologies and sequence analysis of the internal transcribed spacer (ITS) (Fig. 2C and D). The ITS region (including the 5.8S rRNA gene) and the 26S rRNA gene D1/D2 domains were amplified with the primer pair of pITS-F (5′-GTCGTAACAAGGTTAACCTGCGG-3′) and pITS-R (5′-CCTCCGCTTATTGATATGC-3′).^[3]^ Sequence similarity searches were performed using basic local alignment search tool (BLAST), which revealed a complete (100%) match with *S aurantiacum*. In vitro susceptibility testing was performed by a broth microdilution assay according to the methods of the Clinical and Laboratory Standards Institute (CLSI) M38-A2.^[4]^ The minimum inhibitory concentration (MIC) of amphotericin B, itraconazole, posaconazole, voriconazole, caspofungin, and micafungin for the isolate determined at 48 hours were 16, 16, 2, 1, 8, and 8 μg/mL, respectively.

One week after the third operation, conjunctival suturing was again performed because of dehiscence of the sutured site with tissue melting. While hospitalized, the patient was treated with topical voriconazole 2% hourly, 0.125% amphotericin B every 4 hours, and intravenous voriconazle 4 mg/kg every 12 hours, based on the susceptibility of the isolated pathogen to antifungal agents. After 9 weeks of systemic voriconazole treatment, intravenous for 3 weeks and oral for 6 weeks, the infection was controlled and did not recur or worsen.

## 3 Discussion

Species of the genus Scedosporium include *Scedosporium prolificans*, *Indiella americana*, and complexes of *Scedosporium boydii* (*S aurantiacum*, *Pseudallescheria minutispora*, *S dehoogii*, *Scedosporium dehogii*, and *Pseudallescheria boydii*).^[5]^*S apiospermum* and *S prolificans* have been considered the main Scedosporium pathogens in the human body. *S prolificans* was reported to be the most virulent species. *S aurantiacum*, which causes severe infection, is as virulent as *S prolificans*, with strain-specific differences in virulence observed in a mouse model.^[6]^

Previous reports described the risk factors of Scedosporium species infection were diabetic cornea erosion that subsequently infected by fungus from contaminated water,^[7]^ a history of trauma especially caused by vegetation (wooden stick, sugar cane leaf),^[8,9]^ and cataract surgery.^[9]^ Although the patient described in this report was immunocompetent and did not have diabetes, her regional infection was not controlled by long-term treatment with antifungal agents.

Patients with *Scedosporium* keratitis have been treated with topical agents, including voriconazole alone or combined with other antifungal agents.^[7,8]^*Scedosporium* species, however, are generally resistant to antifungal agents, a patient with refractory fungal keratitis by *S apiospermum* with diabetes treated with topical Amphotericin B and Voriconazole over 6 weeks, rapid surgical debridement did a role in complete healing of her cornea, final visual acuity was 6/9.^[7]^ In a case series of 10 patients, 9 patients were treated with topical natamycin 5%, Topical voriconazole 1% was added for 3 patients, of whom 1 patient was given oral fluconazole 200 mg and 1 patient was given oral ketoconazole 400 mg. Outcome data were available for 9 patients: 1 patient was lost to follow-up before completion of treatment; 8 patients responded to the medical therapy, while 1 patient progressed to corneal perforation necessitating penetrating keratoplasty.^[8]^ In one case series including 11 keratitis and 2 sclerokeratitis, both cases presenting with sclerokeratitis were unresponsive to medico-surgical treatment, progressing to panophthalmitis and evisceration.^[9]^

*S aurantiacum* may have greater virulence than *S apiospermum*, suggesting that longer treatment with voriconazole (in patients with susceptible isolates) and early surgical intervention may be helpful in treating the former. Enucleation would better eliminate all infected issues than evisceration, as the latter is associated with a risk of necrotic scleritis and poor prognosis, as in the present patient.

The clinical course of fungal keratitis, especially response to antifungal agents, is dependent on the infecting species.^[10]^ Culture findings are limited in identifying organisms, whereas sequencing of polymerase chain reaction-amplified DNA is good for accurate and rapid identification of species and can help optimize treatment.

## Author contributions

**Conceptualization:** Hyuna Kim, Ja-Young Ahn, Seong-Jae Kim

**Data curation:** Hyuna Kim, Ja-Young Ahn, Woong-Sun Yoo, Seong-Jae Kim

**Formal analysis:** Hyuna Kim, Ja-Young Ahn, Seong-Jae Kim.

**Investigation:** Hyuna Kim, Ja-Young Ahn, Seong-Jae Kim

**Methodology:** Hyuna Kim, Jong Hee Shin.

**Resources:** Ja-Young Ahn, Jong Hee Shin

**Supervision:** Seong-Jae Kim.

**Validation:** Ja-Young Ahn, In-Young Chung, Seong-Wook Seo, Seong-Jae Kim.

**Visualization:** Ja-Young Ahn, Jong Hee Shin

**Writing – original draft:** Hyuna Kim, Ja-Young Ahn.

**Writing – review & editing:** Hyuna Kim, In-Young Chung, Seong-Wook Seo, Woong-Sun Yoo, Jong Hee Shin, Seong-Jae Kim.
